# Atlas of Venereal and Skin Diseases

**Published:** 1889-07

**Authors:** 


**Atlas of Venereal and Skin Diseases.** Comprising original illustrations and selections from the plates of Prof. M. Kaposi, of Vienna; Mr. J. Hutchinson, of London; Prof. J. Neuman, of Vienna; Profs. A. Fournier and Hardy, and Drs. Ricord, Cullebrier, and Vidal, of Paris; Prof. Leloir, of Lisle; Dr. Unna, of Hamburg; Dr. Silva Aranjo, of Rio Janeiro; Dr. P. A. Morrow, of New York; Dr. E. L. Keyes, of New York; Dr. A. R. Robinson, of New York; Dr. J. Nevins Hyde, of Chicago; Dr. Henry G. Piffard, of New York, and others. With original text by PRINCE A. MORROW, A. M., M. D.; Clinical Professor of Venereal Diseases; formerly Clinical Lecturer on Dermatology in the University of the City of New York; Surgeon to Charity Hospital, etc. New York: William Wood & Co. 1889. J. H. Matteson, Buffalo, N. Y. Fasciculi XIV. and XV.

We have, in previous numbers of the JOURNAL, favorably noticed this work. The numbers, as they appear, maintain the high character aimed at in their preparation and publication.

The list of plates in the fourteenth fasciculus are as follows:

Plate LXVI.—Lupus Erythematosus, Lupus Vulgaris.
Plate LXVII.—Lupus Papillaris. Tuberculosis Papillomatosa Cutis.
Plate LXVIII.—Sarcoma of Trunk, Sarcoma of Face.
Plate LXIX.—Epithelioma, Rodent Ulcer.
Plate LXX.—Leprosy.

The list of plates in the fifteenth fasciculus are as follows:

Plate LXXI.—Scabies.
Plate LXXII.—Pediculosis Corporis.
Plate LXXIII.—Chromophytosis.
Plate LXXIV.—Tricophytosis and Favus.
Plate LXXV.—Eczema Marginatum Favus.

The text in each number, from the pen of Dr. Morrow, gives a brief and clear description of the disease illustrated in the plates. The work is a most valued aid to the practitioner in the study of a class of cases otherwise very obscure. We commend it, and also congratulate the publishers on the success of their efforts to furnish the profession with the best aid possible in this important department of medicine.

